# Antiretroviral protease inhibitors induce features of cellular senescence that are reversible upon drug removal

**DOI:** 10.1111/acel.13750

**Published:** 2022-12-20

**Authors:** Chisaka Kuehnemann, Jun‐Wei B. Hughes, Pierre‐Yves Desprez, Simon Melov, Christopher D. Wiley, Judith Campisi

**Affiliations:** ^1^ Buck Institute for Research on Aging Novato California USA; ^2^ University of Southern California Los Angeles California USA; ^3^ Jean Mayer USDA Human Nutrition Research Center on Aging Tufts University Boston Massachusetts USA; ^4^ California Pacific Medical Center San Francisco California USA

**Keywords:** aging, antiretroviral drug, human immunodeficiency virus, inflammation, senescence‐associated secretory phenotype

## Abstract

Antiretroviral drugs have dramatically improved the prognosis of HIV‐infected patients, with strikingly reduced morbidity and mortality. However, long‐term use can be associated with signs of premature aging. Highly active antiretroviral therapy generally comprises two nucleoside reverse transcriptase inhibitors (NRTIs), with one of three additional antiretroviral drug classes, including protease inhibitors (PIs). One commonality between mitochondrial dysfunction (induced by NRTIs) and defects in lamin A (induced by PIs) is they can cause or accelerate cellular senescence, a state of essentially irreversible growth arrest, and the secretion of many bioactive molecules collectively known as the senescence‐associated secretory phenotype (SASP). We hypothesized that senescent cells increase following treatment with certain HIV therapies. We compared the effects of two distinct HIV PIs: ritonavir‐boosted atazanavir (ATV/r) and ritonavir‐boosted darunavir (DRN/r), used in combination treatments for HIV infection. Upon ATV/r, but not DRN/r, treatment, cells arrested growth, displayed multiple features of senescence, and expressed significantly upregulated levels of many SASP factors. Furthermore, mice receiving sustained ATV/r treatment showed an increase in senescent cells and age‐related decline in physiological function. However, removing treatment reversed the features of senescence observed in vivo and cell culture. Given how these features disappeared with drug removal, certain features of senescence may not be prognostic as defined by an irreversible growth arrest. Importantly, for patients that are treated or have been treated with ATV/r, our data suggest that switching to another PI that does not promote premature aging conditions (DRN/r) may improve the associated age‐related complications.

## Graphical abstract

Patients infected with HIV are at increased risk for multiple diseases of aging. Here, Kuehnemann et. al. show that treating cells and mice with protease inhibitors (PIs) used in antiretroviral therapies for HIV induces many characteristics of cellular senescence. Surprisingly, senescent phenotypes triggered by PI treatment were reversed after removal of the drug, and treated mice regained lost physiological function. These findings suggest that intermittent treatment or switching to therapies that do not cause senescence could reduce PI‐induced premature aging.

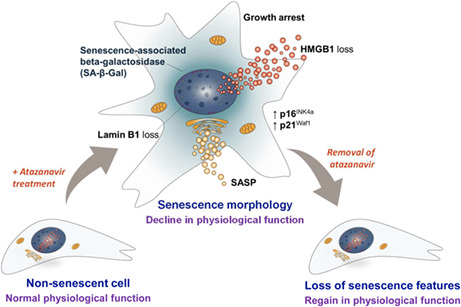

**Abbreviations**
3MR: trimodal reporter
AIDS: acquired immunodeficiency syndrome
ART: antiretroviral therapies
ATR/r: atazanavir/ritonavir
DAPI: 4′,6‐diamidino‐2‐phenylindole
DMSO: dimethyl sulfoxide
DOXO: doxorubicin
DRV/r: darunavir/ritonavir
EdU: 5‐Ethynyl‐2'‐deoxyuridine
ER: endoplasmic reticulum
GSE22: p53 genetic suppressor element 22
HAART: highly‐active antiretroviral therapy
HGPS: hutchinson‐Guilford progeria syndrome
HIV: human immunodeficiency virus
HSV‐TK: herpes simplex virus thymidine kinase
LMNA: lamin A
LUC: luciferase
mRFP: monomeric RFP
NRTI: nucleoside reverse transcriptase inhibitor
PI: protease inhibitor
SA‐B‐gal: senescence‐associated beta‐galactosidase
SASP: senescence‐associated secretory phenotype

## 1 INTRODUCTION

Aging is a major risk factor for the development of many chronic diseases. It is characterized by a loss of physical integrity, leading to a progressive decline in cellular and tissue function over time. This decline increases the risks for many age‐related diseases, including atherosclerosis, heart failure, sarcopenia, renal failure, osteoporosis and neurodegenerative diseases such as Alzheimer's and Parkinson's diseases (Campisi, 2013). The diversity of these diseases, and their co‐morbidity over time, suggests that some basic biological processes might underlie age‐related pathologies. One way to identify such processes is to study conditions that accelerate aging phenotypes and pathologies. In humans, this approach is afforded by the unexpected side effects of treatments for two life‐threatening diseases: genotoxic anticancer therapies and anti‐retroviral therapies (ART). The latter is comparatively understudied, despite its rising clinical importance and potential for uncovering new mechanisms of aging.

Clinical studies show that HIV‐infected patients have on average a 10‐year shorter life expectancy relative to those without infection (Lohse et al., 2007). That deficit shrinks to only 8‐years with access to healthcare and timely initiation of ART (Marcus et al., 2016). Several factors in HIV patients might influence lifespan and susceptibility to chronic diseases. Low level viral replication, toxic side effects of ART medications and chronic immune activation have all been implicated as contributing factors (Pathai et al., 2014). The individual influence of ART on disease susceptibility has been difficult to parse out, given that these drugs are essential for HIV+ patients' long‐term survival, and uninfected individuals have not taken ART drugs until the more recent introduction of pre‐exposure prophylaxis.

Antiretroviral guidelines set by the U.S. Department of Health and Human Services for the treatment of HIV infection in adults and adolescents recommend highly active antiretroviral therapy (HAART) for the treatment of HIV infection. A HAART regimen is generally a combination of two nucleoside reverse transcriptase inhibitors (NRTIs), with a third inhibitor from one of three antiretroviral drug classes including an integrase strand transfer inhibitor, a nonnucleoside reverse transcriptase inhibitor (NNRTI), or a protease inhibitor (PI) (Shafer & Vuitton, 1999). This clinical approach suppresses HIV and prevents the development and transmission of AIDS. However, accumulating evidence from cell culture and mouse models of ART treatment implicate PIs in the pro‐aging side effects of ART.

PIs such as lopinavir, atazanavir, and ritonavir inhibit activity of the mammalian protease ZMPSTE24 (Coffinier et al., 2008). ZMPSTE24 processes the major nuclear matrix protein lamin A (LMNA). A sporadic dominant mutation in ZMPSTE24 causes the rare human premature aging disorder Hutchinson‐Gilford progeria syndrome (HGPS/Progeria). Children with progeria accumulate an unprocessed form of LMNA, termed progerin, and die generally in the second decade of life, primarily of cardiovascular disease; however, the children also suffer from lipodystrophy, type 2 diabetes, and bone and skin fragility (Maraldi et al., 2011; Olive et al., 2010). Further, HGPS cells are more susceptible to cellular senescence in culture than their normal counterparts (Mu et al., 2020). Notably, normal individuals also produce progerin, which increases in multiple tissues throughout life, albeit at much lower levels (Olive et al., 2010). Thus, PIs might accelerate pro‐aging processes by acting on molecular pathways that drive natural aging. One such process is cellular senescence.

Cellular senescence is a complex stress response featuring a persistent growth arrest coupled to resistance to apoptosis and a multifaceted secretory phenotype, termed senescence‐associated secretory phenotype (SASP) (Rodier & Campisi, 2011). The SASP includes numerous factors, including pro‐inflammatory cytokines, proteases and growth factors (Acosta et al., 2013; Coppe et al., 2008). Cellular senescence was originally described as the finite proliferative capacity of normal human fibroblasts in culture (Hayflick, 1965). More recent studies indicate that senescence also mediates multiple physiological and pathological processes, including embryonic development (Munoz‐Espin et al., 2013; Storer et al., 2013), wound healing (Demaria et al., 2014), tissue repair (Krizhanovsky, Yon, et al. 2008), and aging (Baker et al., 2011).

Here, we show that the PI atazanavir, boosted with ritonavir (ATV/r), induces features of senescence which, surprisingly, are reversible upon removal of the drug. Cells cultured in the presence of ATV/r exhibit features of cellular senescence includingupregulated senescence‐associated β‐galactosidase (SA‐β‐gal) activity, increased expression of the tumor suppressors p16^INK4a^ and p21^WAF1^, decreased LMNB1 expression, reduced nuclear high mobility group B1 (HMGB1), reduced EdU incorporation, and significantly upregulated levels of SASP factors. Upon ATV/r removal, however, many cells resumed proliferation. T Furthermore, mice receiving sustained ATV/r treatment showed increased features of senescence and deficits in function, including decreased heart function. When ATV/r was removed, mice regained physiological heart function, commensurate with a loss of senescence markers. Since these features are used to identify senescent cells in vivo, our results suggest that certain features of senescence may not be prognostic of cellular senescence as defined by irreversiblegrowth arrest. Biomedically, these results matter for patients who are taking/have taken HIV protease inhibitors (PIs)—going off these medications and/or switching to another PI that does not promote premature aging conditions (e.g., DRN/r).

## 2 RESULTS

### 2.1 The protease inhibitor (PI) atazanavir induces senescence features in cultured human cells

To examine the effects of HIV PIs on senescent phenotypes, we treated human IMR‐90 fibroblasts in culture with atazanavir boosted with ritonavir, as administered in patients. Ritonavir, although itself a PI, is generally used as a pharmacokinetic enhancer in combination therapies to increase the effectiveness of HIV medicines. As fibrosis‐associated genes were found elevated in HIV patients taking anti‐retroviral therapies (ART) (Kusko et al., 2012), we treated the cells with clinically relevant doses: 10–20 μM each (Auclair et al., 2014) of atazanavir + ritonavir (ATV/r).

**FIGURE 1:**
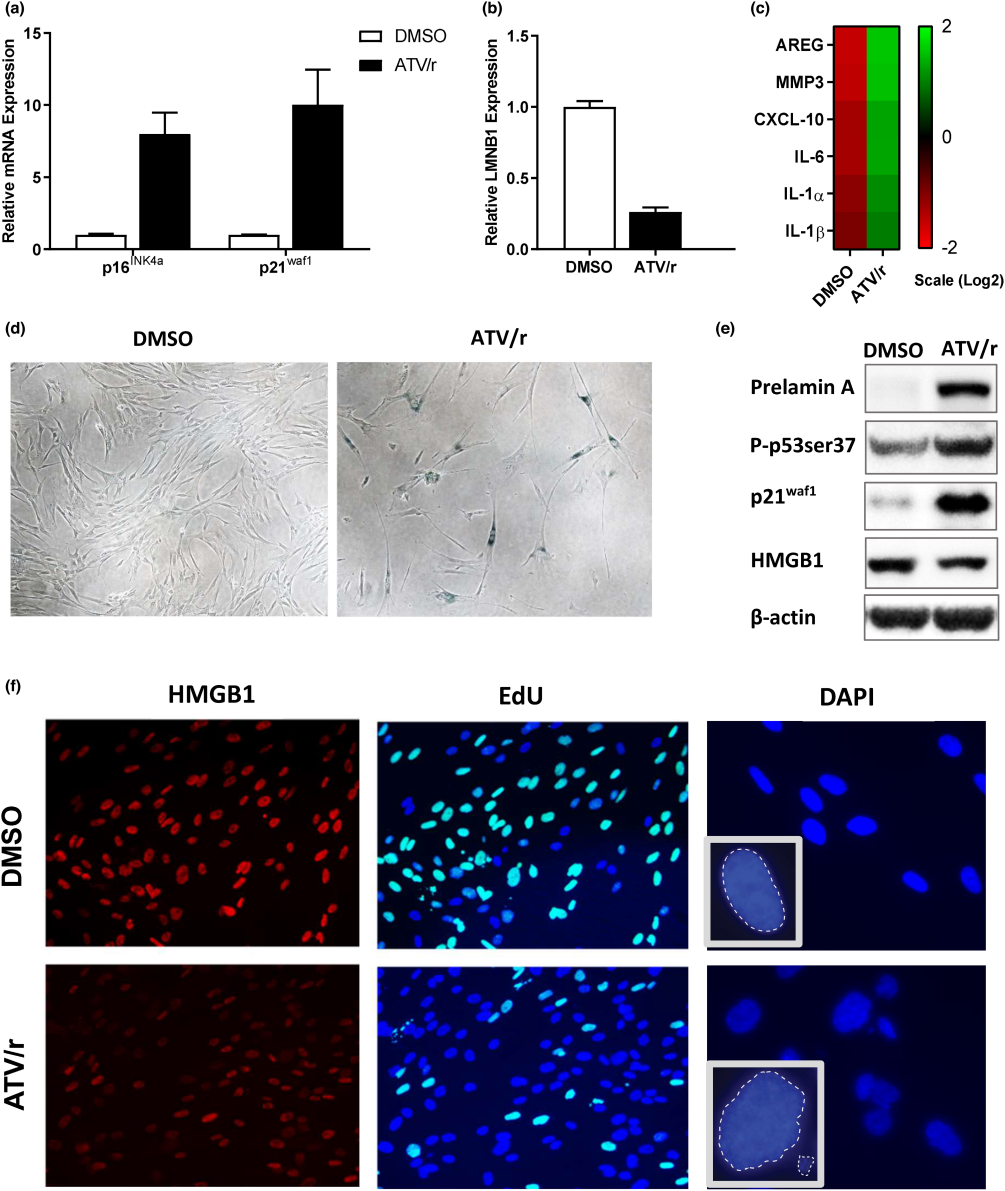
The anti‐HIV PI cocktail atazanavir + ritonavir (ATV/r) induces senescence in cultured cells. IMR‐90 primary human fibroblasts were cultured in the presence of ATV/r for 14 days. (a). RNA was isolated from untreated (DMSO) and ATV/r‐treated cells, and p16^INK4a^ and p21^WAF1^ mRNA levels, normalized for Actin mRNA, were measured by qPCR. (b). mRNA levels of LMNB1 in DMSO‐ and ATV/r‐treated cells were similarly measured. (c). SASP component mRNA levels were measured using qPCR. (d). Representative images of SA‐β‐gal positivity in control cells (left panel) and cells induced to senesce by ATV/r (right panel). (e). Intracellular levels of proteins prelamin a, activated (P‐ser37) p53, p21^WAF1^, HMGB1 and beta‐Actin (control) using western analysis. (f). Cells were analyzed for HMGB1 release from the nucleus, proliferation (EdU), and nuclei morphology (DAPI) by microscopy.

ATV/r‐treated cells showed arrested growth and several features of senescence including increased expression of the tumor suppressors p16^INK4a^ and p21^WAF1^ (Figure 1a), decreased LMNB1 expression (Figure 1b) and significantly upregulated levels of the SASP factors such as AREG, IL‐6, CXCL‐10, IL‐1α, IL‐1β and MMP3 (Figure 1c). MMPs are known to promote inflammation by processing cytokines, such as IL‐1β, and stimulating leukocyte infiltration (Parks et al., 2004). Treated cells also exhibited a flat morphology and upregulated senescence‐associated β‐galactosidase (SA‐β‐gal) activity (Figure 1d). Western analyses showed increased protein levels of prelamin A and growth arrest markers, phospho‐p53 and p21^WAF1^ (Figure 1e). Further, immunofluorescence staining showed reduced nuclear high mobility group B1 (HMGB1), an alarmin that initiates an inflammatory response (Davalos et al., 2013), and reduced EdU incorporation indicative of a decrease in cell proliferation. Nuclear staining also showed abnormal nuclear architecture in ATV/r‐induced senescent cells, including dysmorphic nuclear blebbing (Figure 1f), a characteristic of laminopathies. However, ATV/r‐treated cells did not stain positive for the DNA damage markers 53BP1 and ᵧH2AX (Figures S1‐S6).

**FIGURE 2:**
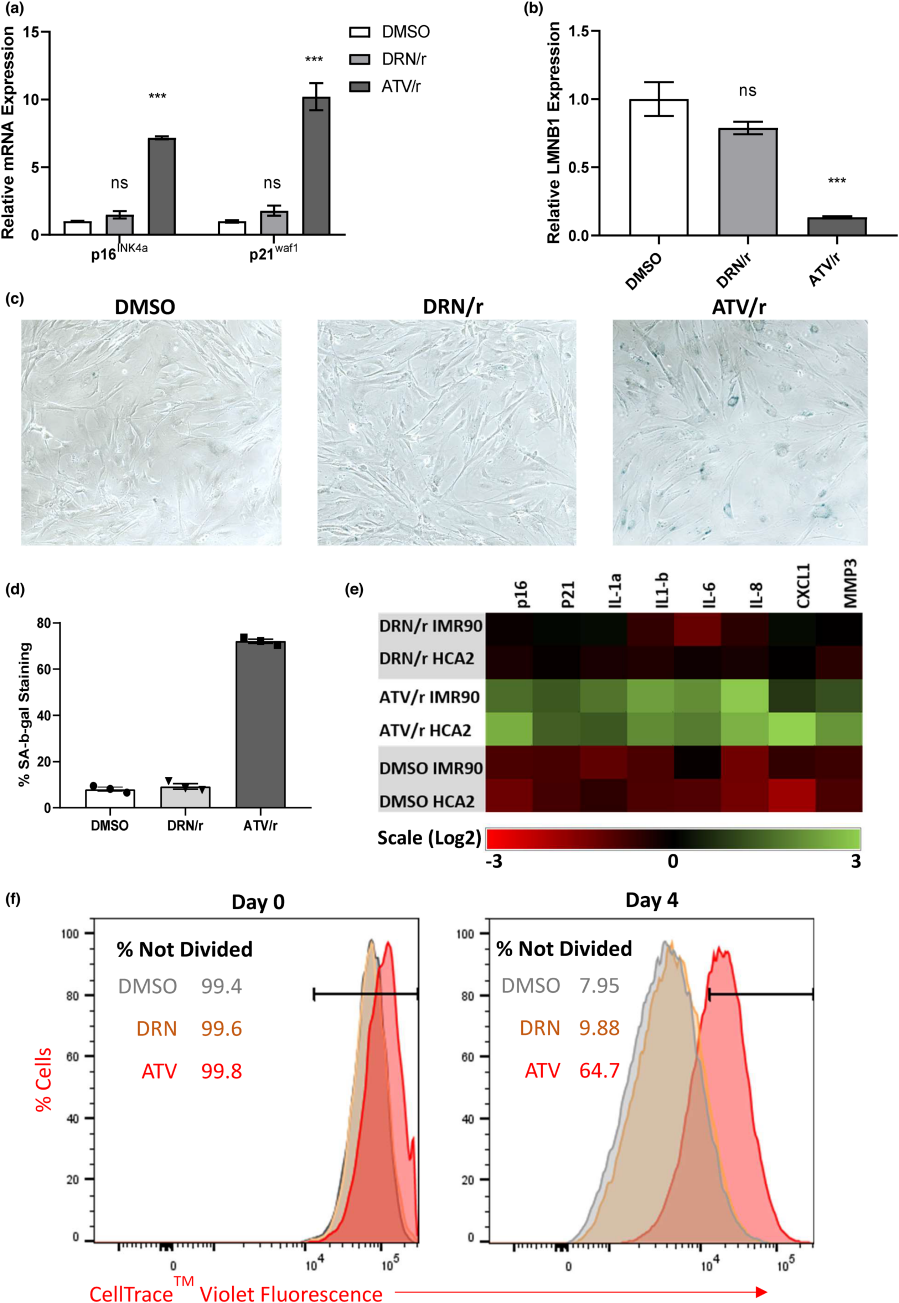
The HIV PI darunavir does not induce senescence in culture. Mouse p16‐3MR dermal fibroblasts were treated with DMSO (control), ATV/r or DRN/r for 14 d prior to analyses. (a). mRNA levels of p16^INK4a^ and p21^WAF1^ were measured by qPCR. (b). mRNA levels of LMNB1 in treated cells as per (a). (c). Representative images of SA‐β‐gal positivity in control cells (DMSO), and DRN/r‐ and ATV/r‐treated cells. (d). Quantification of SA‐β‐gal positivity. (e). SASP component mRNA levels were measured by qPCR in the indicated cell populations. (f). Cell proliferation of DMSO‐, DRN/r‐ and ATV/r‐treated cells. Cell proliferation was followed for 4 days using the CellTrace™ violet reagent.

We next determined the effects on senescent phenotypes of darunavir boosted with ritonavir (DRN/r), another commonly prescribed HIV PI combination. Although several PIs, including atazanavir, are implicated in pro‐aging effects through inhibition of the metalloproteinase ZMPSTE24, which cleaves farnesyl‐prelamin A to mature unfarnesylated lamin A (LMNA) (Young et al., 2005), Darunavir does not inhibit ZMPSTE24, and consequently does not cause an accumulation of prelamin A (Coffinier et al., 2008) or cellular senescence (Auclair et al., 2014). Importantly, DRN/r has not been associated with age‐related phenotypes and pathologies associated with ART, and has a more favorable safety profile compared to ATV/r (Arathoon et al., 2013; Menzaghi et al., 2013; Orkin et al., 2013). Mouse dermal fibroblasts treated with DRN/r did not arrest growth or exhibit features of senescence. DRN/r, unlike ATV/r, did not upregulate the expression of p16^Ink4a^ or p21^Waf1^ and did not downregulate LMNB1 expression (Figure 2a,b). Senescent MDF cells were killed by ABT263 in a dose‐dependent manner (Figure S2). DRN/r‐treated cells did not stain positively for SA‐β‐gal activity (Figure 2c,d), did not express common SASP proteins (Figure 2e), and continued to proliferate as determined by CellTrace™ Violet dye dilution (Figure 2f). Finally, lopinavir, another PI that inhibits ZMPSTE24 (Coffinier et al., 2008), also induced features of senescence such as upregulation of p16^INK4a^ and p21^WAF1^ and downregulation of LMNB1, and upregulated levels of SASP factors such as AREG, IL‐6, CXCL‐1, IL‐1α, IL‐1β and MMP1 (Figure S3). These findings suggest that anti‐HIV PIs that target ZMPSTE24 are responsible for the pro‐aging effects of ART and act by inducing senescence.

### 2.2 ATV/r‐treated cells undergo a p53‐dependent growth arrest

**FIGURE 3:**
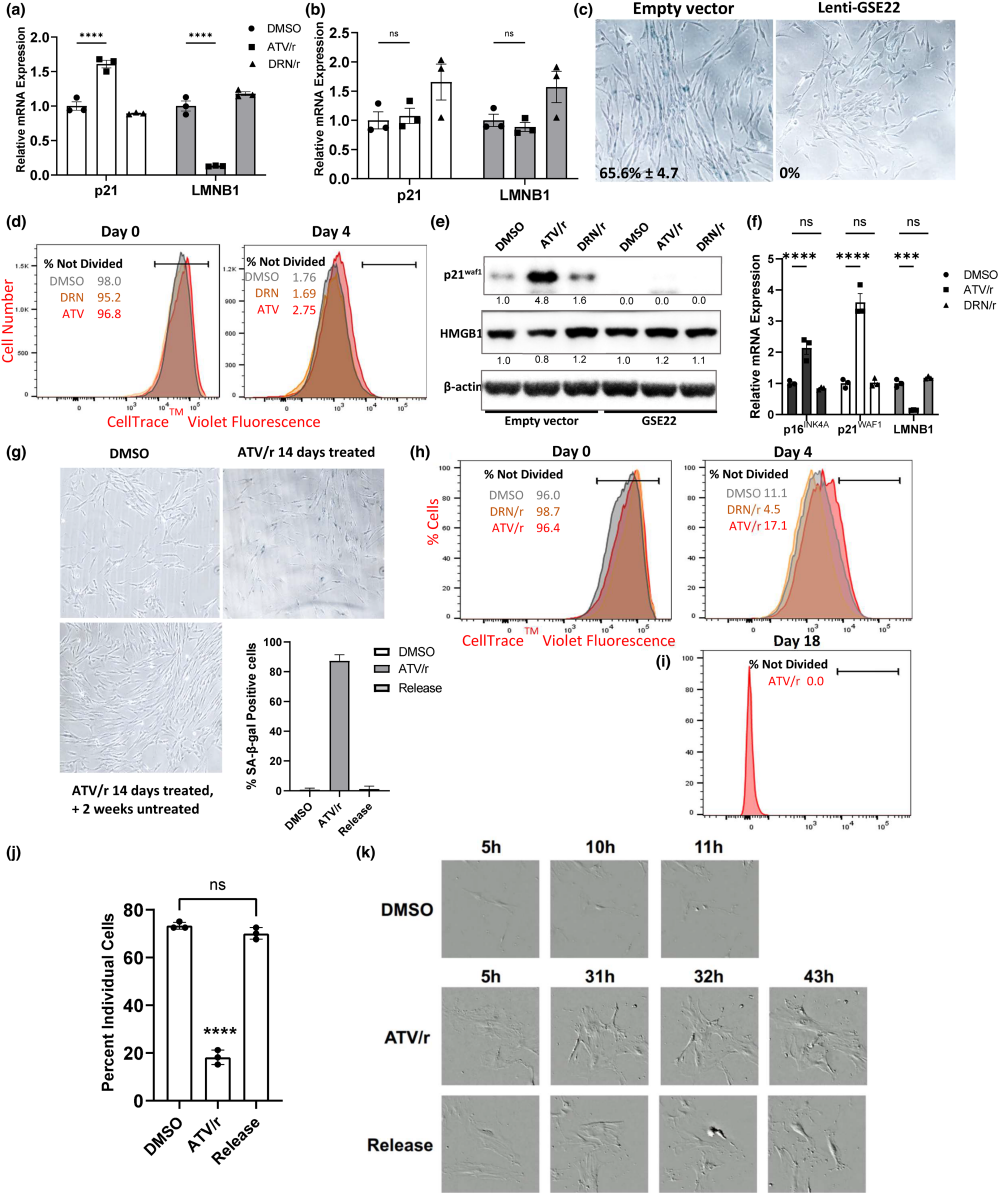
ATV/r‐induces a p53‐dependent growth arrest that is reversed upon drug removal. IMR‐90 fibroblasts were transduced with lentiviral vectors expressing GSE22, a peptide that inactivates p53, or no insert (vector), and cultured in the presence of ATV/r or DRN/r for 14 days. (a). p21^WAF1^ and LMNB1 mRNA levels in control (vector) cells were measured by qPCR. (b). p21^WAF1^ and LMNB1 mRNA levels in GSE‐transduced cells were measured by qPCR. (c). Representative images of SA‐β‐gal positivity in control (vector) or GSE22‐transduced cells treated with ATV/r. (d). Cell proliferation of DMSO‐, ATV/r‐, and DRN/r‐treated (Lenti‐GSE22‐transduced) IMR‐90 cells was analyzed. Cell proliferation was followed for 4 days using the CellTrace™ violet reagent. (e). Western blot confirming p53 inactivation in GSE22‐transduced cells by absence of p21^WAF1^ expression and retained nuclear HMGB1. (f). IMR‐90 fibroblasts were treated with DMSO, ATV/r or DRN/r for 14 d prior to analysis. p16^INK4a^, p21^WAF1^ and LMNB1 mRNA levels in the three cell populations. (g). Representative images and quantification of SA‐β‐gal positivity in DMSO‐treated, ATV/r‐treated or ATV/r‐released cells. (h). Proliferation of DMSO‐, DRN/r‐ and ATV/r‐treated cells was followed for 4 days using the CellTrace™ violet reagent. (i). the 17.1% non‐dividing ATV/r‐treated cells were re‐plated and cultured for 2 weeks without ATV/r, then re‐analyzed for proliferation. (j). Percentage of individual cells from DMSO‐ or ATV/r‐treated cultures that had divided by 96 h. (k). Images of individually tracked DMSO‐treated, ATV/r‐treated or ATV/r‐released cells. Magnification and scale are consistent between treatment groups.

As p53 often initiates the senescence growth arrest, and we observed increased phosphorylation of p53‐S37 (Figure 1e), we assessed the p53 dependence of ATV/r‐induced senescence. We used human IMR‐90 fibroblasts expressing a genetic suppressor element (GSE22), a peptide that prevents p53 tetramerization and causes inactive monomeric p53 to accumulate (Gudkov et al., 1993; Ossovskaya et al., 1996). We transduced IMR‐90 fibroblasts with lentiviral vectors expressing GSE22 or no insert (vector) and cultured in the presence of ATV/r or DRN/r for 14 days. Compared to empty vector transduced cells treated with ATV/r or DRN/r (Figure 3a), GSE22 suppressed the induction of senescence by ATV/r, as determined by unchanged levels of p21^WAF1^ and LMNB1 (Figure 3b). Similarly, a relatively low efficiency of p53 shRNA knockdown reduced expression of p21^WAF1^ compared to control cells (Figure S4). While empty vector control cells stained for SA‐β‐gal activity following ATV/r treatment, GSE22‐infected cells did not (Figure 3c). GSE22‐transduced cells treated with ATV/r continued to proliferate similarly to control cells and/or cells treated with DRN/r (Figure 3d). Western blotting confirmed that GSE22, but not the empty vector, reduced p21^WAF1^ levels in ATV/r‐treated cells, as expected for loss of p53 activity. Further, GSE22 retained nuclear HMGB1 following ATV/r treatment (Figure 3e). Thus, p53 activity is necessary for ATV/r‐induced senescence growth arrest.

### 2.3 Removal of ATV/r from cells in culture reverses features of senescence

To assess the persistence of ATV/r‐induced senescence, we cultured IMR‐90 fibroblasts with ATV/r for 14 days, and assessed senescence markers. We then removed the drugs and maintained the cultures for another 14 days before analysis. As expected, IMR‐90 cells treated with ATV/r for 14 days arrested growth and showed increased expression of p16^INK4a^ and p21^WAF1^, and loss of LMNB1 expression, compared to vehicle (DMSO) or DRN/r‐treated cells (Figure 3f). However, when ATV/r was removed, and cultures maintained for another 14 days without subculturing, surprisingly, senescent phenotypes reversed. SA‐β‐gal positivity declined to ~1% compared to ~87% after the initial 14 day‐treatment (Figure 3g). Unlike clonal expansion, which results in the formation of colonies that arise from individual cells that escape senescence, cell division was diffuse and relatively uniform across the ATR/r washout cultures (Figure 3g), consistent with reversal of cell cycle arrest.

One way senescent cells contribute to aging phenotypes is through the senescence growth arrest. This loss of proliferative capacity can prevent progenitor cells from repopulating a tissue, leading to loss of tissue function (Zhou et al., 2008). We assessed proliferation by ATV/r‐treated cells, alongside vehicle DMSO‐ and DRN/r‐treated cells as proliferating controls. We followed cell proliferation during the last 4 days of drug treatment – on day 10, the CellTrace™ Violet dye was added to a replicate plate and a baseline measured using the reagent. Notably, the degree to which ATV/r induced senescent phenotypes varied among cell types, with mouse fibroblasts being more sensitive to ATV/r‐induced senescence growth arrest compared to IMR90 human fibroblasts in the same time frame (Figures 2f, 3h). Labeled IMR‐90 cells were gated at day 0 to encompass >95% of events and the same gates were used at days 4 and 18 to ascertain the frequency of undivided cells. After 4 days, 17.1% of ATV/r‐treated cells remained arrested (Figure 3h). This cell population was sorted and returned to culture for 2 weeks without ATV/r treatment, after which it was re‐analyzed for proliferation. The cells had undergone multiple cell divisions by day 18, with no cells falling within the day 0 gate. These data suggest that all or most cells, once growth arrested, had re‐entered the cell cycle upon drug removal (Figure 3i). Thus, ATV/r induces a growth arrest that is p53‐dependent and has many features of senescence, but is reversible upon the removal of the drugs.

Finally, we conducted cell tracking experiments in which cells from DMSO‐ or ATV/r‐treated cultures were plated at low density and individual cells tracked for 96 hours or until confluence prevented tracking. While DMSO‐treated cells divided more quickly than ATV/r‐released cells, there were no differences in the percentages of initially seeded cells that had divided by 96 hours, whereas only a low percentage of continuously treated ATV/r cells divided during this time (Figure 3j). Further, ATV/r‐released cells retained their size and senescent morphology until after cell division (Figure 3k). After 2 weeks without treatment, SA‐β‐gal positivity was ~4% in the ATV/r‐released cell cultures compared to ~83% in cell cultures continuously treated with ATV/r. Thus, loss of features of senescence in these cells was not due to colony expansion, but reversal upon drug removal.

### 2.4 Mice given ATV/r show accelerated aging phenotypes

To determine if some PIs promote aging phenotypes in mice, we analyzed p16‐3MR transgenic mice in which the promoter of the tumor suppressor p16^Ink4a^ is used to track a portion of senescent cells (Demaria et al., 2014). The promoter drives expression of the 3MR (trimodality reporter) fusion protein containing functional domains of a synthetic *Renilla* luciferase (LUC), monomeric red fluorescent protein (mRFP), and truncated herpes simplex virus 1 (HSV‐1) thymidine kinase (HSV‐TK) (Wang et al., 2004). LUC allows the detection of 3MR‐expressing cells by luminescence.

**FIGURE 4:**
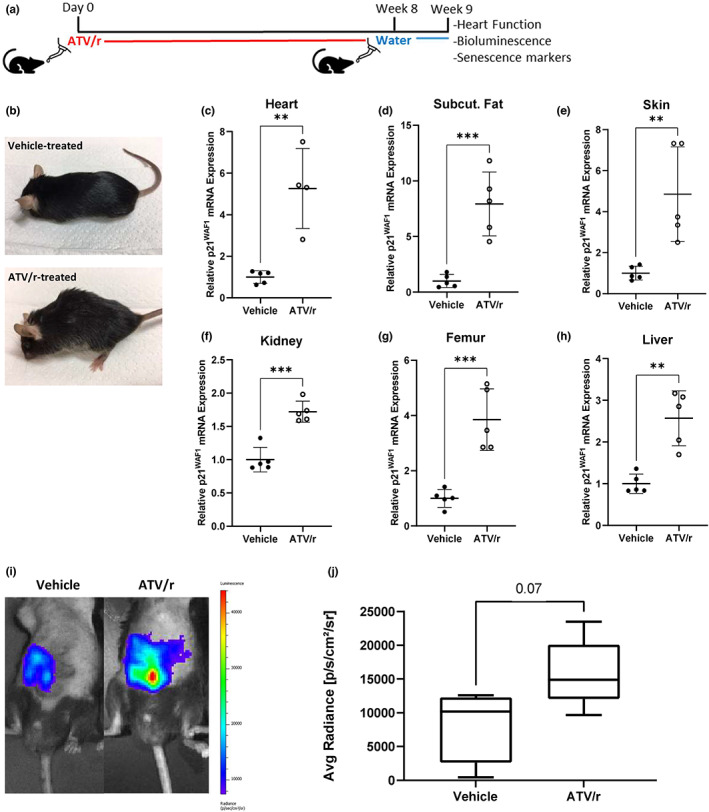
ATV/r treatment accelerates aging phenotypes. Young adult mice at 5 months of age were treated with 62 mg/kg atazanavir and 21 mg/kg ritonavir in drinking water for 8 weeks. (a). Schematic of the experimental setup. (b). Representative phenotypic differences between ATV/r‐treated mice and age‐matched controls are shown. (c–h). p21^Waf1^ mRNA levels were measured by qPCR in various tissues. (i). Representative images of p16‐3MR male mice injected with coelenterazine and measured for luminescence using the Xenogen imaging system. (j). Quantification of luminescence presented in I.

Young adult p16‐3MR mice were administered ATV/r at clinically relevant doses (62 mg/kg atazanavir, 21 mg/kg ritonavir), normalized for mouse body surface area (Reagan‐Shaw et al., 2008) for 8 weeks (Figure 4a). We then performed functional assays and collected tissues for senescence analyses. Mice treated with ATV/r exhibited an aged appearance, including gray hair, hair loss and localized loss of fat tissue (lipodystrophy) (Figure 4b). These phenotypes are common among patients on long‐term ART and those with genetic defects in LMNA (Caron et al., 2007). Tissues from the heart, subcutaneous fat, kidney, bone, skin and liver showed significantly increased expression of p21^Waf1^ (Figure 4c–h). However, there were no significant differences in body weight comparing vehicle‐ and ATV/r‐treated mice (Figure S5). Finally, p16^Ink4a^ expression was more modestly increased in some tissues (Figure S6), consistent with the luminescence measurements: ATV/r caused a small increase in whole‐body bioluminescence (Figure 4i,j), comparable to the increased *p16* ^*Ink4a*^ mRNA in tissues. These results suggest that ATV/r induces features of cellular senescence in vivo in mice.

### 2.5 Senescent cells appear at sites of ATV/r‐induced pathology

**FIGURE 5:**
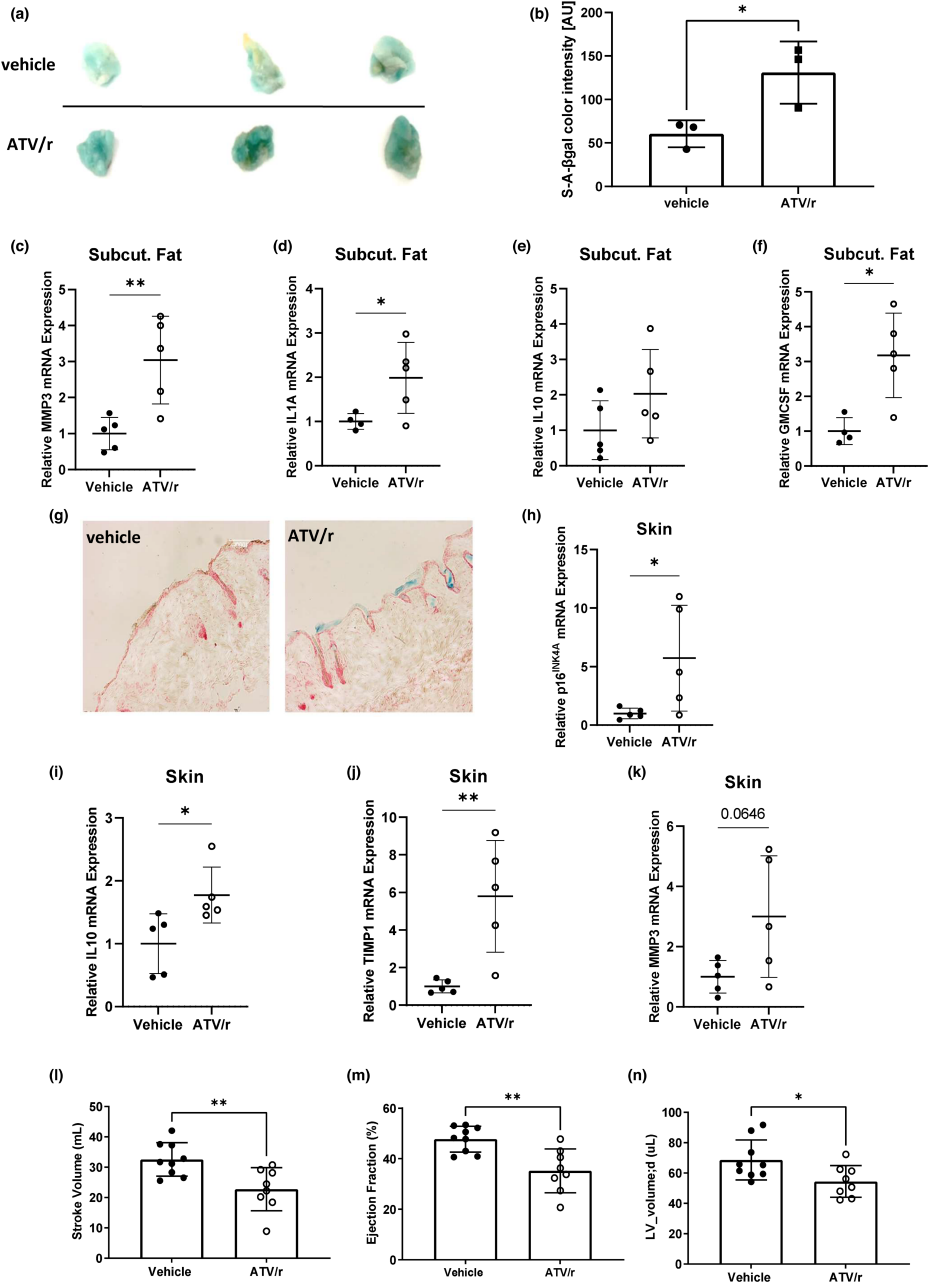
ATV/r‐treated mice accumulate senescent cells at sites of age‐related pathologies. Young adult mice (5 months of age) were treated with 62 mg/kg atazanavir and 21 mg/kg ritonavir in drinking water for 8 weeks. (a). Representative images of SA‐β‐gal staining in subcutaneous fat from vehicle and ATV/r‐treated mice. (b). Quantification of SA‐β‐gal staining presented in a. (c–f). selected SASP factors (MMP3, IL‐1a, GMCSF and IL‐10) and p21^Waf1^ mRNA expression levels were measured in subcutaneous fat by qPCR and normalized to Actin. (g). Representative images of sectioned dorsal skin stained for SA‐β‐gal activity. (h). qPCR analysis for senescence marker p16^Ink4a^. (i–k). mRNA expression levels of some SASP markers from skin (IL‐10, TIMP1 and MMP3) were measured by qPCR and normalized to Actin. (l–n). cardiac activity was measured by echocardiography.

Mice treated with ATV/r exhibited localized loss of fat tissue/lipodystrophy, reduced bone density and reduced cardiac activity, despite no overall loss in body weight. Histochemical analysis of subcutaneous fat revealed more SA‐β‐gal staining in ATV/r‐compared to vehicle‐treated animals (Figure 5a,b). In subcutaneous fat, ATV/r‐treated mice exhibited core features of the SASP, including *Mmp3*, *IL1a*, *Gmcsf* and *Il10*, and showed increased *p21* ^*Waf1*^ expression (Figure 5c–f). This pro‐inflammatory SASP has been shown to occur in humans after genotoxic chemotherapy (Coppe et al., 2008; Sun et al., 2012), and chronic inflammation contributes to several age‐related pathologies (Chung et al., 2009; Franceschi & Campisi, 2014).

Dorsal skin from mice treated with ATV/r similarly stained positive for SA‐β‐gal in the stratum corneum (Figure 5g) and showed significantly increased *p21* ^*Waf1*^ and *p16* ^*Ink4a*^ expression compared to vehicle‐treated mice (Figures 4e, 5h). Further, expression of the SASP factors, *Il10*, *Timp1* and *Mmp3*, was higher compared to vehicle‐treated mice (Figure 5i–k). Echocardiography revealed significant reductions in stroke volume, ejection fraction and ventricular volume (Figure 5l–n) in ATV/r‐treated mice. Other parameters measured included End Diastolic Volume, End Systolic Volume, Fractional Shortening, Global Longitudinal Strain, End Diastolic Left Ventricular Mass, and End Systolic Left Ventricular Mass, although there were no significant differences between vehicle‐ and ATV/r‐treated mice (data not shown).

### 2.6 Cessation of ATV/r treatment improves senescence and aging phenotypes

**FIGURE 6:**
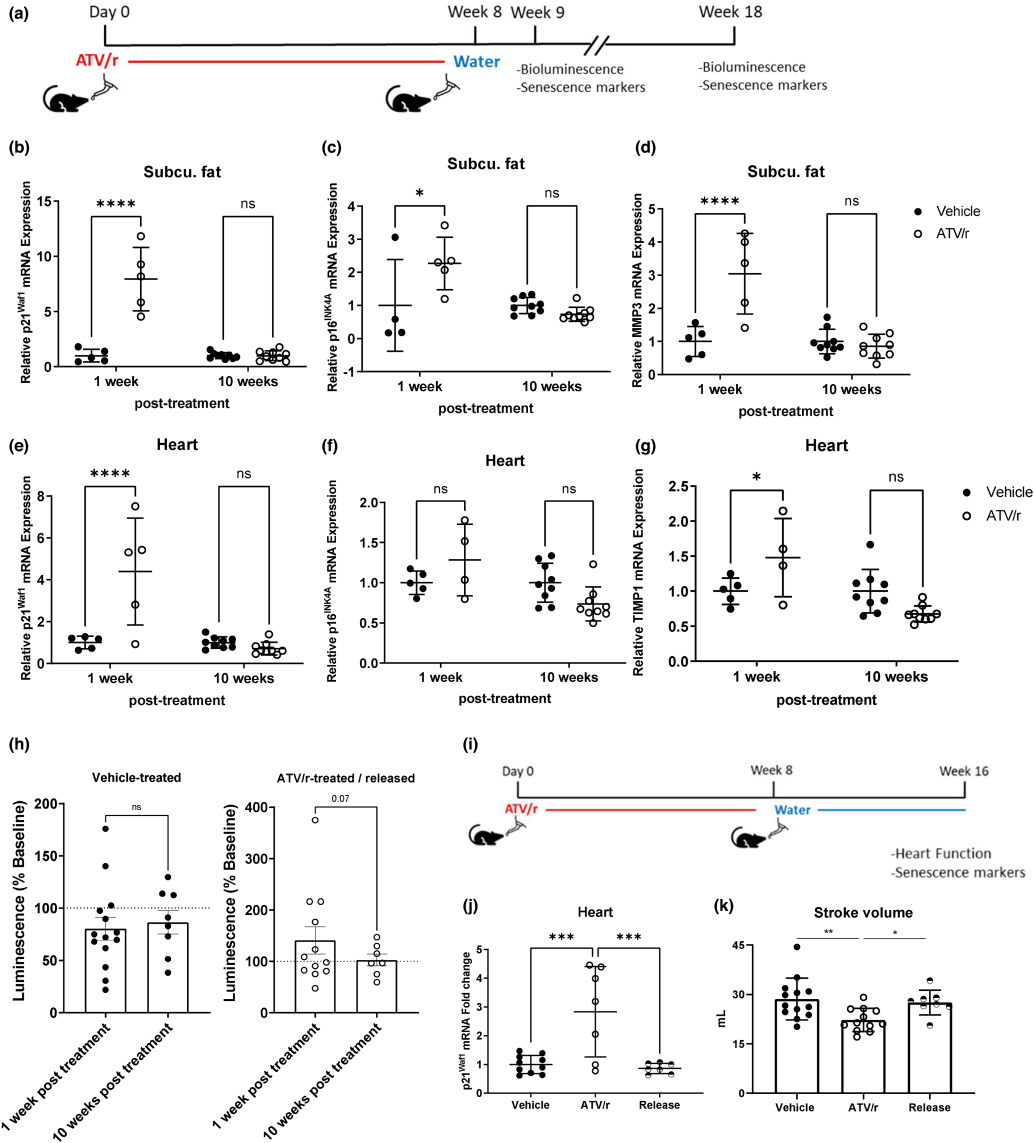
Removal of ATV/r treatment reverses senescent phenotypes in mice and improves age‐related phenotypes. Young adult mice (5‐months old) were treated with 62 mg/kg atazanavir and 21 mg/kg ritonavir in drinking water for 8 weeks. (a). Schematic of the experimental setup. (b,c). p21^Waf1^ and p16^Ink4a^ mRNA levels in and subcutaneous fat were measured by qPCR. (d). SASP factor MMP3 mRNA levels in the subcutaneous fat tissue were measured by qPCR and normalized to Actin. (e,f). p21^Waf1^ and p16^Ink4a^ mRNA levels in heart tissue were measured by qPCR. (g). SASP factor TIMP1 mRNA levels in the heart were measured by qPCR and normalized to Actin. (h). p16‐3MR male mice were injected with coelentarazine, and luminescence was measured at baseline, after 8 weeks of treatment, and 10 weeks post‐treatment. (i). Schematic of the experimental setup. (j). qPCR analysis for p21^Waf1^ mRNA levels in heart tissue. (k). Cardiac activity was measured by echocardiography.

To determine whether ATV/r removal could reverse aging phenotypes associated with its administration in mice, we gave mice ATV/r or vehicle (DMSO) for 8 weeks, and then removed the drug for 10 weeks, after which we assessed physiological function and collected tissues for analyses (Figure 6a). Mice receiving ATV/r for 8 weeks showed the expected increase in senescent phenotypes, as determined by increased expression of p16^Ink4a^ and p21^Waf1^ and ATV/r‐induced pathology, such as subcutaneous fat. However, 10 weeks after ATV/r removal, *p16* ^*Ink4a*^ and *p21* ^*Waf1*^ expression were similar in ATV/r‐ and vehicle‐treated mice (Figure 6b,c). Further, the SASP factor *Mmp3*, initially elevated after 8 weeks of treatment, declined following 10 weeks without treatment (Figure 6d). We found similar results in heart tissue (Figure 6e–g). Whole body luminescence moderately increased upon ATV/r treatment and decreased upon drug removal (Figure 6h), suggesting an accumulation of p16‐driven senescent cells by ATV/r treatment that is lost upon drug removal.

In mice treated with ATV/r for 8 weeks (Figure 6i), p21^Waf1^ expression increased in the heart, then decreased 8 weeks after drug removal (Figure 6j). ATV/r‐treated mice also exhibited deficits in heart function, including stroke volume, which were reversed 8 weeks after ATR/r was removed (Figure 6k). At that time, mice regained heart function and more closely resembled normal vehicle‐treated mice. Thus, cessation of ATV/r treatment in mice reverses some of the drug‐induced aging phenotypes and pathologies.

## 3 DISCUSSION

Increased access to antiretroviral medication has improved worldwide survival in HIV‐infected patients, with a 39% decline in AIDS‐related deaths since 2010. In 2019, 38 million people worldwide were living with HIV. Of these, 67% accessed treatment, and most had undetectable levels of the virus. However, long‐term antiretroviral use has been associated with serious side effects resembling accelerated aging, resulting in reduced lifespans for HIV‐infected patients receiving ART compared to uninfected individuals (Lohse et al., 2007). Indeed, the use of antiretroviral drugs has been related to adverse events that can compromise patients' health. Senescent cells might contribute to the toxic side effects of two drug classes: (1) the NRTIs, which can inhibit mtDNA POLG, resulting in mitochondrial dysfunction, and (2) the PIs, some of which inhibit the protease ZMPSTE24, impairing maturation of the major nuclear protein lamin A (LMNA). Genetic studies also suggest that ZMPSTE24 plays a role in the endoplasmic reticulum (ER), clearing clogged translocons, and in the unfolded protein response (UPR), which could explain an alternative pathway to senescence as chronic ER stress has been implicated in dysfunctional DNA repair and decreased proliferation (Dufey et al., 2020).

The PI combinations ATV/r and DRN/r are commonly prescribed to treat patients with HIV. Studies comparing the efficacy of ritonavir‐boosted atazanavir or darunavir showed similar effectiveness, but with different tolerability. DRV/r is generally preferred over ATV/r (Lennox et al. 2014) owing to higher toxicity rates associated with ATV/r over DRV/r. The U.S. Department of Health and Human Services HIV treatment guidelines now recommend DRV/r as the preferred option for initiating combination antiretroviral therapy and has reclassified ATV/r as an alternative/second‐line therapy. Patients switching treatment to DRV/r are less likely to experience side effects from the drugs, and thus can better adhere to their treatment compared to those changing to combinations with ATV/r or lopinavir/ritonavir (LPV/r), another treatment option for HIV patients (Antoniou et al., 2017). Their differential induction of senescence may explain the differences in toxicity. The effect on senescent phenotypes, of removing/switching these drugs has not been explored, and for this reason we evaluated these two PIs.

Here we show that (i) treatment with ATV/r results in features of senescence, including a SASP, in relevant human and mouse cell types in culture, (ii) ATV/r‐treated cells undergo a p53‐dependent growth arrest that is ultimately reversible, (iii) mice administered ATV/r show accelerated aging phenotypes, (iv) features of senescence appear at sites of ATV/r‐induced pathology, and (v) cessation of ATV/r treatment reverses these senescence features.

Upon treatment with ATV/r, we show multiple features characteristic of senescent cells that are not prognostic of irreversible growth arrest. These data raise questions about the accuracy of using these features as biomarkers of senescent cells if we define senescence by irreversible growth arrest. Since these features are used to identify senescent cells in vivo, and here we show we can reproduce many of these features without irreversibility, perhaps some of these features do not necessarily mark cells that are irreversibly growth arrested. Comparing outcomes between the senescence characteristics imposed by treatment with doxorubicin (doxo), a chemotherapeutic drug known to induce senescence (Demaria et al., 2017), and that imposed by ATV/r, we see that many senescence features, including the growth arrest induced after doxo treatment, endure long after the drug is removed. Doxo induces DNA damage, which results in irreversible growth arrest, whereas ATV/r appears to be a transient stressor that induces senescence features that are reversible once removed. In agreement with this, ATV/r‐treated cells did not stain positive for DNA damage markers.

We first attributed the reversibility of ATV/r phenotypes in mice to immune clearance (immune surveillance), as immune cells attracted by the SASP can remove nearby damaged cells (Krizhanovsky, Yon, et al. 2008). Also, premalignant senescent hepatocytes in mice are reported to induce a T‐cell‐mediated adaptive immune response (Krizhanovsky, Xue, et al. 2008). Since both doxo‐induced senescent cells (Demaria et al., 2017) and injected senescent cells (Xu et al., 2017) persist for weeks‐to‐months, whereas ATV/r‐induced senescent cells do not, it is unlikely they are cleared by the immune system. Furthermore, we reproduced similar results in culture using normal human fibroblasts. Because this reversal was observed in homogenous human fibroblast cultures in the absence of immune cells, it could not be attributable to immune surveillance.

To link PIs and LMNA defects to specific age‐related pathologies driven by senescent cells, we used the p16‐3MR mouse model (Demaria et al., 2014). However, unlike aged mice accumulating high levels of p16‐positive cells, we found that senescent cells accumulating upon PI‐treatment expressed more *p21* ^*Waf1*^, whereas *p16* ^*Ink4a*^ was only modestly increased. Transgenic p16‐3MR or similar INK‐ATTAC (Baker et al., 2016) mice, allow for selective elimination of p16‐positive senescent cells. However, this approach is limited to diseases in which p16‐positive cells are major drivers of senescence‐related pathologies, which did not appear to be the case in our ATV/r model. New inducible p21‐Cre mouse models could shed light on how senescent cells significantly expressing p21^Waf1^ influence PI‐related aging pathologies (Binsheng Wang 2021).

Senolytics, which selectively kill senescent cells, are emerging as promising aging therapies. The senolytic drug ABT‐263 induces apoptosis in senescent cells expressing either p16^INK4a^ or p21^WAF1^. Certain illness, such as pulmonary fibrosis, can be reversed by senolytic drugs (Pan et al., 2017), and early stage clinical trials are underway to use senolytics in treating various diseases (Kirkland & Tchkonia, 2020). Senolytics might therefore be useful for future research linking ART drugs to age‐related diseases driven by senescent cells. However, our results call into question the use of some features of senescence as biomarkers of senescence in vivo; some of these biomarkers may be a stress response that has features of senescence. Ultimately, regardless of how these features are prognostic for long‐term senescence, there is medical relevance for patients treated with ATV/r. Adopting an intermittent treatment strategy informed by when adverse effects become evident with treatment or switching therapies may improve the age‐related complications associated with HIV PI treatment and ultimately improve outcomes for HIV‐infected patients.

## 4 EXPERIMENTAL PROCEDURES

### 4.1 Cell culture and treatment

IMR‐90 primary human lung fibroblasts (ATCC; #CCL‐186) were cultured in Dulbecco's Modified Eagle Medium (Thermo Fisher Scientific; #12430–054) supplemented with penicillin and streptomycin (5000 U/ml and 5000 μg/ml) (Thermo Fisher Scientific; #15070063) and 10% fetal bovine serum (Thermo Fisher Scientific; #2614079). Mouse dermal fibroblasts were isolated from the dorsal skin of 3‐month‐old mice as described (Demaria et al., 2014). Primary mouse cells were expanded for no more than 10 doublings. HCA2 human fibroblasts were obtained from O. Pereira‐Smith (The University of Texas Health Science Center). All cell types were maintained at 37°C, 10% CO_2_ and 3% O_2_.

### 4.2 Drugs and induction of senescence

Atazanavir, ritonavir and darunavir were from Medchem Express. Cells were treated in appropriate media for 14 days with DMSO (control) or drugs dissolved in DMSO at 10–20 μM (Cmax plasma concentrations) according to the literature (Auclair et al., 2014). Cells were then washed with PBS and placed in serum‐free DMEM. Cells and conditioned media were collected after 24 h for analyses.

### 4.3 Vectors

To inactivate p53, we used the genetic suppressor element GSE22 as described (Beausejour et al., 2003; Gudkov et al., 1993; Ossovskaya et al., 1996).

### 4.4 RT‐PCR

Cells were incubated with drugs for 14 days. RNA was isolated from cells using the Bioline Isolate II RNA Mini Kit. RNA was isolated from homogenized tissues using TRIzol reagent (Thermo Fisher Scientific) with the Direct‐zol RNA MiniPrep Kit (Genesee Scientific) as recommended by the supplier. mRNA was extracted, followed by reverse transcription (Capacity cDNA Reverse Transcription Kit, #4368814, Life Technologies). cDNA synthesis and qRT‐PCR were performed as described (Demaria et al., 2017) using the LightCycler 480 (see tables for the primers and probes used).

### 4.5 Mouse model

p16‐3MR mice were maintained in the AALAC‐accredited Buck Institute for Research on Aging animal facility. All procedures were approved by the Institutional Animal Care and Use Committee. p16‐3MR mice were bred in house. For ATV/r‐induced senescence, 4‐5‐month old p16‐3MR mice were administered 62 mg/kg atazanavir and 21 mg/kg ritonavir in drinking water for 8 weeks.

### 4.6 Echocardiography

Two‐dimensional (2D) transthoracic echocardiographic analysis was performed using a high frequency (20–46 MHz) Visualsonics Vevo 2100 micro‐ultrasound system with the echocardiography MS‐400 transducer (Visualsonics). Individual mice were placed on a heating pad (37°C) and anesthetized with isoflurane at concentration of 1.5–2%. Data acquisition was performed B‐, and M modes, from parasternal long axis view. Left ventricle volumes, diameters and wall thicknesses were calculated using VevoStrain software (Visualsonics).

### 4.7 Bioluminescence

For cell cultures, a Renilla Luciferase Assay System (Promega) was used according to the manufacturer's protocol, measuring luminescence with a luminometer. For in vivo studies, mice were injected i.p. with 15 μg of Xenolight RediJect Coelentarazine h (Calipers). 25 min later, the mice were anesthetized with isofluorane, and luminescence measured using the Xenogen IVIS‐200 Optical In Vivo Imaging System at 5 min medium binning (Caliper Life Sciences).

### 4.8 Western blotting

Cells were washed with cold PBS, lysed, and subjected to SDS‐PAGE using 4%–12% Bis‐Tris gels. Separated proteins were transferred to polyvinylidene fluoride membranes, which were blocked and incubated overnight at 4°C with anti‐rabbit primary antibodies (p21^WAF1^, Cell Signaling #2947, 1:1000; p‐p53, Cell Signaling #9289, 1:1000; HMGB1, abcam# ab18256, 1:2000; prelamin A, Millipore Sigma #mabt858, 1:500; β‐actin, Sigma‐Aldrich #A2228, 1:10000). Membranes were washed and incubated with horseradish peroxidase‐conjugated (1:5000, Cell Signaling) secondary antibodies for 45 min at room temperature and washed again. Signals were detected by enhanced chemiluminescence.

### 4.9 Senescence‐associated β‐galactosidase (SA‐β‐gal) staining

SA‐β‐gal activity was determined using the BioVision Senescence Detection Kit (#K320‐250). For each condition and replicate, cells were counted and 7000 cells seeded into 8‐well culture slides coated with poly‐lysine (Corning, NY; #354632). After 24 h, the staining assay was performed as per the manufacturer's protocol. For each experiment, approximately 100–150 cells were counted.

### 4.10 Cell proliferation assays

To trace multiple generations of cells in culture using dye dilution and flow cytometry, the CellTrace™ Violet Cell Proliferation Kit was used according to the manufacturers protocol (ThermoFisher Scientific, # C34557). CellTrace™ Violet dye was diluted to a final concentration of 5 μM in PBS in a final volume of 1 ml. Cells were then incubated for 20 min at room temperature, protected from light. To remove free dye, 5 ml of culture medium containing 10% FBS was added to the cells, incubated for 5 min, and then the cells were centrifuged and resuspended in FACS buffer (1x PBS, 5% FBS and 2 mM EDTA).

### 4.11 Flow cytometry analysis

Cells were analyzed using a BD FACS Aria (Becton Dickinson). CellTrace™ Violet was excited with the 405 nm laser and detected with the 450/50 band passfilter. The detector voltage was established using unlabeled controls fully on‐scale, then labeled cells were run to confirm they were fully on‐scale. If >5% of labeled cells were off‐scale, voltage was reduced as needed to bring them on‐scale and re‐acquire unlabeled controls. After voltages were configured, fluorescent Rainbow 6‐Peak Calibration Particles were acquired and mean intensity values from all peaks that were well resolved and fully on‐scale in each detector were recorded. To ensure consistency and enable direct comparison of data collected on separate days, the recorded intensities were used as target values when re‐establishing detector voltages on subsequent days. Data were analyzed using FlowJo_v10.6.2. The stained samples were gated at day 0 to encompass >95% of events, and the same gates were used at day 4 to ascertain the frequency of undivided cells.

### 4.12 Statistical analysis

Statistics were assessed using GraphPad Software. For in vivo experiments with multiple comparisons, one‐way ANOVA and Sidak test for multiple comparisons were used. For pairwise comparisons, data were analyzed using the unpaired two‐tailed Student *t* test. Differences between means were considered significant when values were *p* < 0.05 or lower (*); ns denotes nonsignificant. Data are presented as mean values ± SD for in vivo and cell culture experiments. All culture experiments were replicated at least three times.

## AUTHOR CONTRIBUTIONS

CK, CW and JC designed the experiments; CK and JWBH conducted the experiments; CK, PYD, CW, SM and JC analyzed the data; CK, PYD, JWBH, CW and JC wrote and edited the manuscript.

## CONFLICT OF INTEREST

JC is a founder and stockholder of Unity Biotechnology, which is developing drugs to clear senescent cells. JC and SM are partially supported by ONO Pharmaceuticals to explore senolytic mechanisms. The other authors declare no competing financial interests.

## Supporting information


Figures S1‐S6.

## Data Availability

The authors confirm that the data supporting the findings of this study are available within the article.
