# Supplementary material for: Antiretroviral protease inhibitors induce features of cellular senescence that are reversible upon drug removal
Source: Aging Cell. 2022 Dec 20;22(1):e13750. doi: 10.1111/acel.13750 (PMC9835573; doi:10.1111/acel.13750)
Supplement: Supplementary file 1 — Figures S1‐S6. [file ACEL-22-e13750-s001.docx]

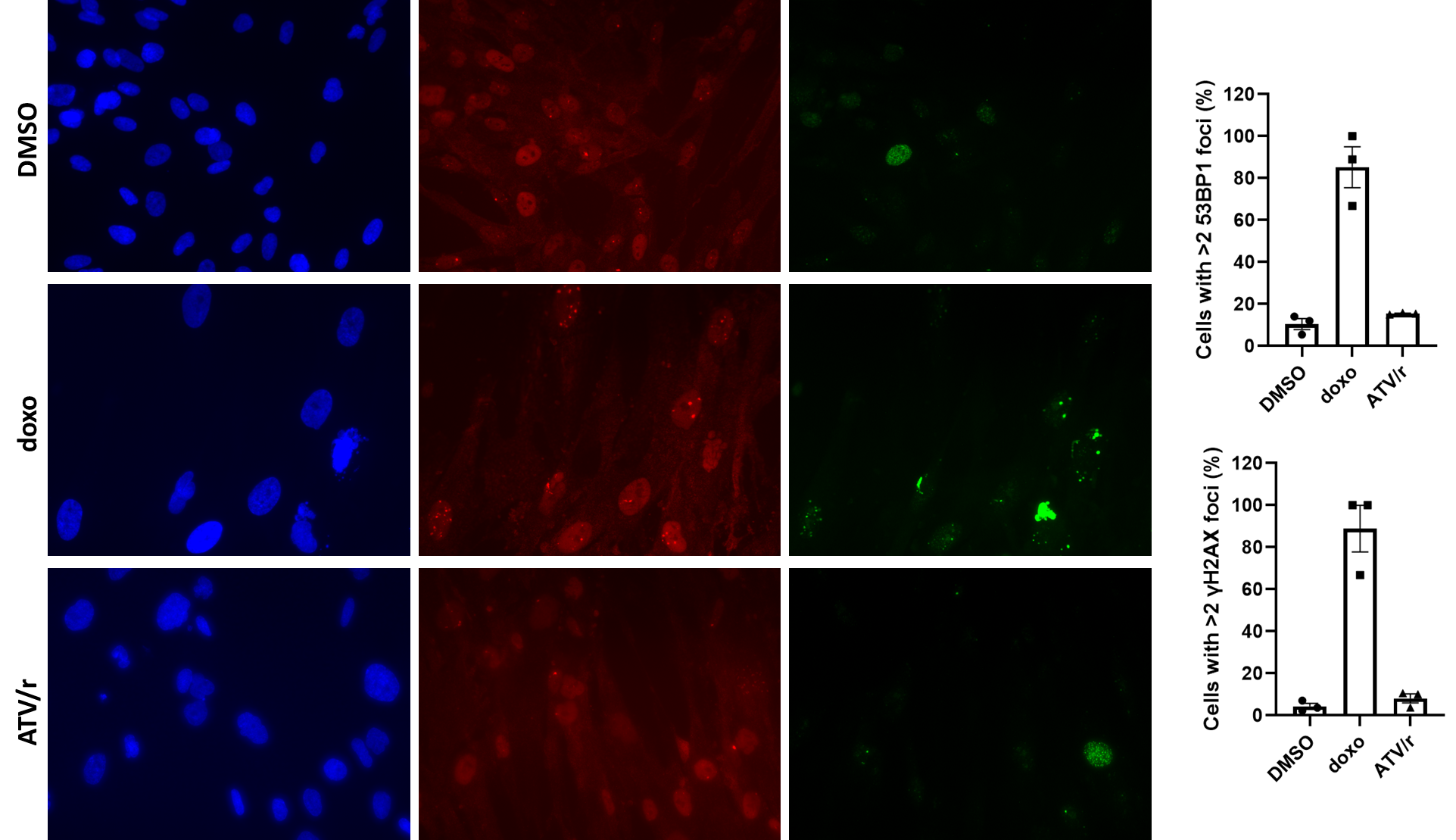


**ᵧH2AX**

**53BP1**

**DAPI**

**Supplementary Figures**

**A**


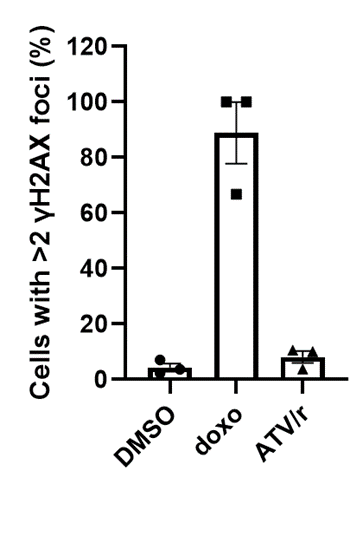

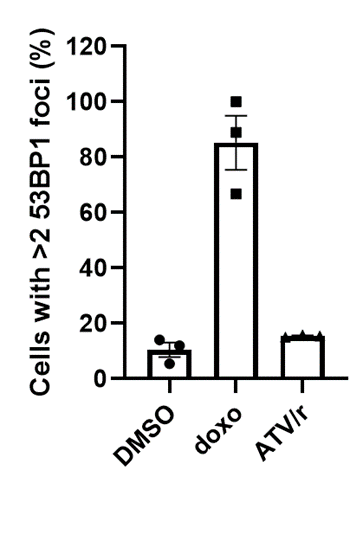


**B**

**C**

**Figure S1. Comparison of 53BP1 and ᵧH2AX staining in ATV/r-treated vs. doxo-treated IMR-90 fibroblasts.** IMR-90 fibroblasts were cultured in the presence of ATV/r for 14 days, or treated with 250nM doxo. **A.** 53BP1 and ᵧH2AX foci in the nuclei were measured in cells induced to senesce by doxo (middle panels) vs. ATV/r-treated cells (bottom panels). Quantification of 53BP1 (**B**) and ᵧH2AX foci (**C**) in DMSO-, doxo- and ATV/r-treated cells.


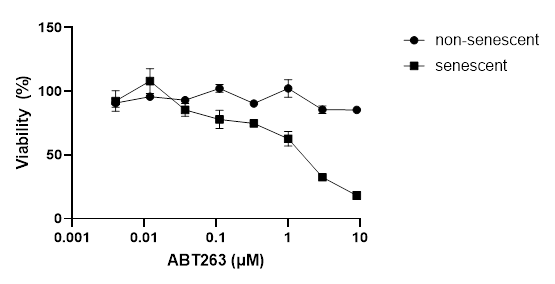


**Figure S2. ABT263 kills cells induced to senescence by ATV/r.** Mouse dermal fibroblasts were cultured in the presence of ATV/r for 14 days then treated with ABT-263 for 3 days. Senescent MDF cells were killed by ABT263 in a dose dependent manner.

**
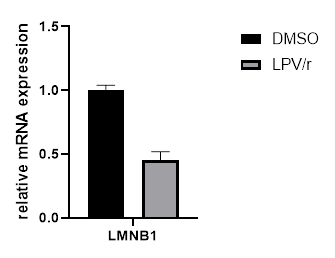
**
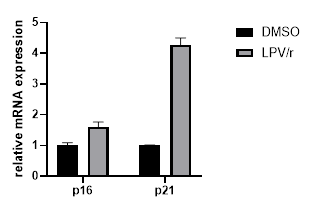


**A**

**B**

**
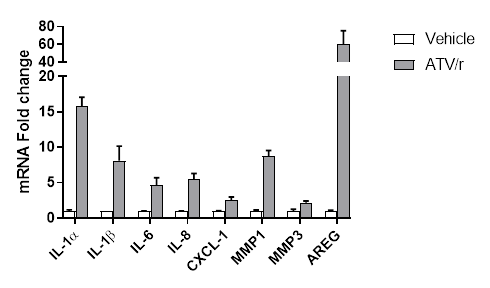
**

**C**

**Figure S3. The anti-HIV PI lopinavir (LPV/r) induces senescence in cultured cells.** IMR-90 primary human fibroblasts were cultured in the presence of LPV/r for 14 days. **A.** RNA was isolated from untreated (DMSO) and LPV/r-treated cells, and p16^INK4a^ and p21^WAF1^ mRNA levels, normalized for actin mRNA, were measured by qPCR. **B.** mRNA levels of *LMNB1* in DMSO- and LPV/r-treated cells were similarly measured. **C.** SASP mRNA levels were measured using qPCR.

**5.6% ± 4.9**

**60.1% ± 5.5**

**A**

**
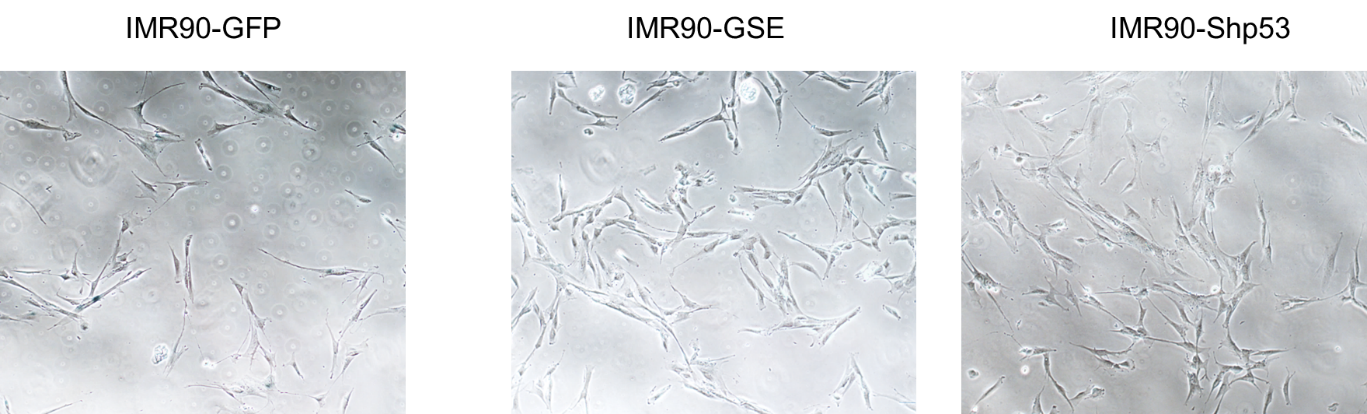

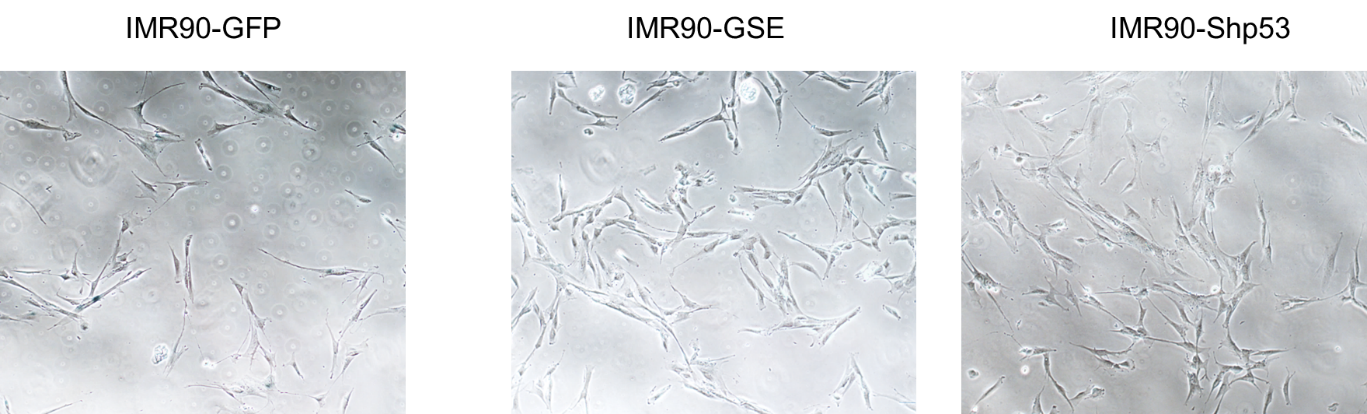
**

**B**

**Figure S4. Assessment of p53 gene knockdown and its effect on ATV/r-induced senescence.** IMR-90 fibroblasts were transduced with lentiviral vectors expressing shp53 or control shGFP and cultured in the presence of ATV/r for 14 days. **A.** Representative images of SA-β-gal positivity in control shGFP (left panel) or shp53 (right panel) cells treated with ATV/r. **B.** p21^WAF1^ mRNA levels were measured by qPCR.


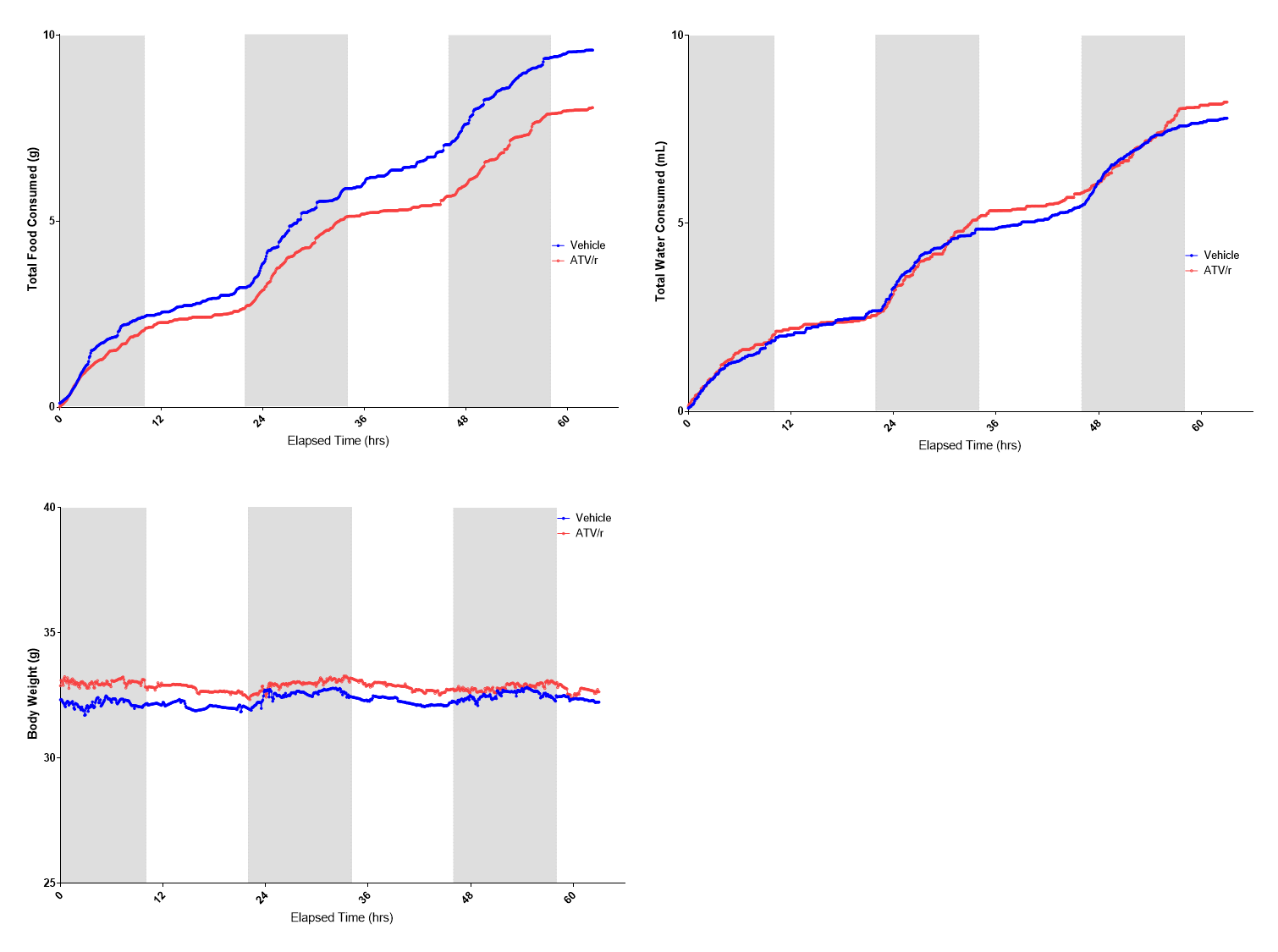


**Figure S5. No significant differences in body weight comparing vehicle- and ATV/r-treated mice.** Body weight of young adult mice (5-month old) treated with vehicle or 62 mg/kg atazanavir and 21 mg/kg ritonavir for 8 weeks.

**A**

**F**

**C**

**B**

**E**

**D**

**Figure S6. ATV/r treatment accelerates aging phenotypes.** Young adult mice at 5 months of age were treated with 62 mg/kg atazanavir and 21 mg/kg ritonavir in drinking water for 8 weeks. **A-F.** p16^Ink4a^ mRNA levels were measured by qPCR in various tissues.
